# Quantifying false positional corrections due to facial motion using SGRT with open‐face Masks

**DOI:** 10.1002/acm2.13170

**Published:** 2021-03-19

**Authors:** Victoria Bry, Anna Laura Licon, James McCulloch, Neil Kirby, Pamela Myers, Daniel Saenz, Sotirios Stathakis, Niko Papanikolaou, Karl Rasmussen

**Affiliations:** ^1^ Department of Radiation Oncology School of Medicine The University of Texas Health Science Center at San Antonio San Antonio TX 78229 USA

**Keywords:** immobilization, radiation therapy, SGRT, SRS, surface guided imaging

## Abstract

**Purpose:**

Studies have evaluated the viability of using open‐face masks as an immobilization technique to treat intracranial and head and neck cancers. This method offers less stress to the patient with comparable accuracy to closed‐face masks. Open‐face masks permit implementation of surface guided radiation therapy (SGRT) to assist in positioning and motion management. Research suggests that changes in patient facial expressions may influence the SGRT system to generate false positional corrections. This study aims to quantify these errors produced by the SGRT system due to face motion.

**Methods:**

Ten human subjects were immobilized using open‐face masks. Four discrete SGRT regions of interest (ROIs) were analyzed based on anatomical features to simulate different mask openings. The largest ROI was lateral to the cheeks, superior to the eyebrows, and inferior to the mouth. The smallest ROI included only the eyes and bridge of the nose. Subjects were asked to open and close their eyes and simulate fear and annoyance and peak isocenter shifts were recorded. This was performed in both standard and SRS specific resolutions with the C‐RAD Catalyst HD system.

**Results:**

All four ROIs analyzed in SRS and Standard resolutions demonstrated an average deviation of 0.3 ± 0.3 mm for eyes closed and 0.4 ± 0.4 mm shift for eyes open, and 0.3 ± 0.3 mm for eyes closed and 0.8 ± 0.9 mm shift for eyes open. The average deviation observed due to changing facial expressions was 1.4 ± 0.9 mm for SRS specific and 1.6 ± 1.6 mm for standard resolution.

**Conclusion:**

The SGRT system can generate false positional corrections for face motion and this is amplified at lower resolutions and smaller ROIs. These errors should be considered in the overall tolerances and treatment plan when using open‐face masks with SGRT and may warrant additional radiographic imaging.

## 1 Introduction

Radiotherapy treatment of tumors in regions of the head and neck require reproducible and accurate techniques. Some stereotactic radiosurgery (SRS) treatments have shifted from the exclusive method of invasive frame‐based treatments to include an additional method using frameless, moldable masks for treatment delivery.^1^ Frame‐based SRS consists of a rigid frame screwed into a patient’s skull for a single fraction of radiation,^2^ while frameless masks consist of a thermoplastic material that is molded to the patient’s face at the time of CT simulation. The clinical goal has focused on improving patient comfort without losing treatment accuracy. The frameless masks have been found to be a practical and reproducible method for image‐guided SRS treatments providing spatial accuracy comparable to frame‐based treatments.^3^ The drawback of the thermoplastic masks is its enclosed method of immobilization that can cause high levels of stress as patients are forced to keep their eyes and mouth closed throughout the treatment process ^4^. The immobilization process can cause anxiety severe enough to disrupt the session,^5^ and patients may experience feelings of fear, anger, or depression. This discomfort has resulted in the introduction of an alternative immobilization method that partially exposes a patient’s face for intracranial^6^ and head and neck^4^ radiotherapy cancer treatments. As a result, claustrophobic patients immobilized with this open‐face style mask have reported less distress during treatment delivery.^4^

Positional uncertainty can negatively impact the accuracy of radiotherapy treatments, so understanding head motion for this immobilization method is important for determining clinical treatment margins.^4^ Head motion, for patients immobilized with closed‐face thermoplastic masks, has generally been characterized based on x‐ray images before and after treatment.^7^ ^,^ ^8^ Open‐face masks allow for real‐time motion monitoring when coupled with surface guided radiation therapy (SGRT) systems both during patient set up^9^ and during treatment delivery.^6^ ^,^ ^10^ ^,^ ^11^ Without the use of radiation, SGRT systems use optical imaging, to generate 3D maps of a patient’s surface. A registration algorithm then compares the live image to a baseline reference image to monitor deviations from original treatment position. Direct imaging of the skin has been considered a more accurate method for motion management as moldable masks may become loose, making small head motion undetectable.^4^ Measurements with phantoms have shown that SGRT is a suitable and reproducible option for intra‐fraction radiosurgery localization.^12^ ^,^ ^13^ An open‐face mask that exposes the entire face has been recommended for anthropomorphic head phantom and patient setup with SGRT.^14^ Several manufacturers produce open face masks, some expose only the patient’s eyes, while others expose the eyes, mouth, and cheeks.

Recent research has aimed to characterize the immobilization performance of using open face masks with SGRT. One study reported that use of a small mask opening or SGRT region of interest (ROI) may generate an apparent shift or false positive with the SGRT system from changes in facial expression (ex. smiling).^4^ This apparent shift demonstrates a potential concern that clinics should consider when using SGRT with frameless open face masks. The primary goal of this work was to demonstrate a previously unexplored area of potential error in SGRT tracking. Our study aims to further quantify false positional shift corrections generated by the SGRT system due to face motion by evaluating multiple SGRT ROIs using two spatial resolution settings. It is our hypothesis that the SGRT system will generate false positional corrections for face motion and they will be amplified at a lower spatial resolution setting and at smaller ROIs. To measure this, human subjects were immobilized using open‐face masks and discrete SGRT ROI were monitored. This experiment recorded positional corrections generated by the SGRT system as human subjects opened and closed their eyes and changed facial expressions to simulate emotions. These methods were performed using two different camera resolutions using the C‐RAD Catalyst HD system.

## 2 Materials and Methods

### 2.A C‐RAD Catalyst HD

**Fig. 1:**
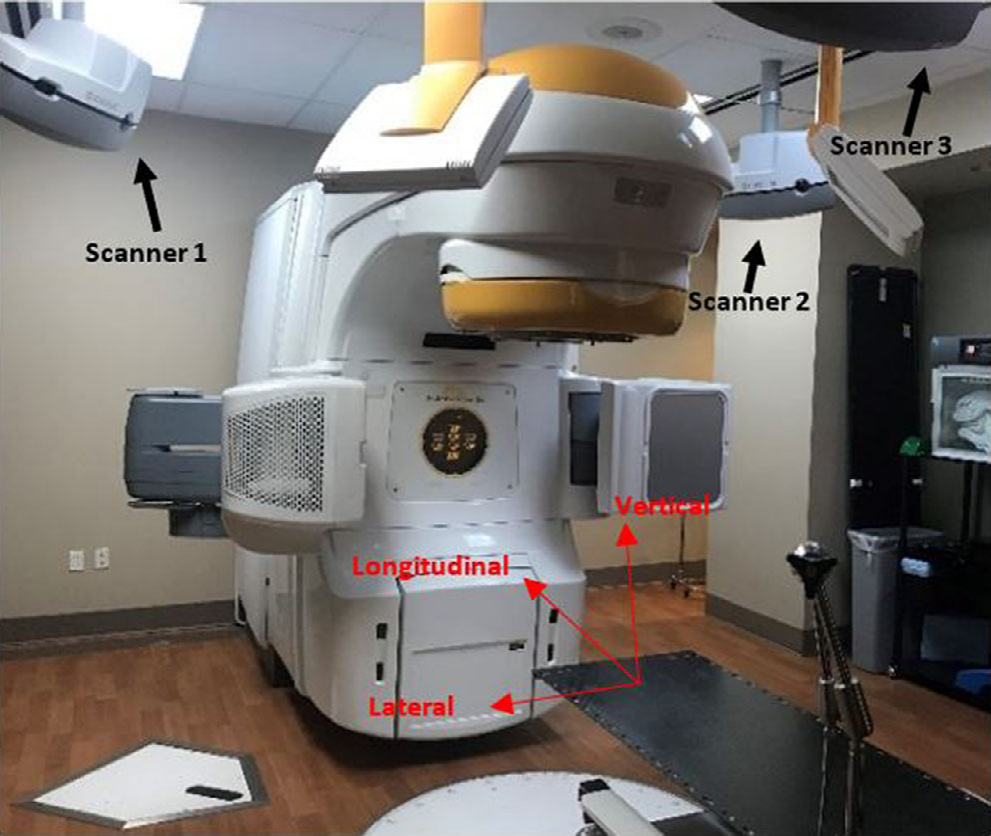
The Catalyst HD (CRAD, Uppsala, Sweden) scanner units set up in the radiation treatment room positioned equidistant above the couch to allow for rotation of the linear accelerator (Novalis TX, Varian, Palo Alto, CA).

The C‐RAD Catalyst HD (C‐RAD, Uppsala, Sweden) uses three ceiling scanner units (see Fig. 1), consisting of a light projector and CCD camera, positioned equidistant above the radiotherapy treatment couch. This system uses a dose‐free triangulation method^15^ to reconstruct a real time surface image of a patient using light patterns. It is used for patient set up, respiratory gating (ex. Deep Inspiration Breath Hold), and real‐time motion management to submillimeter accuracies. A deformable image registration algorithm compares the real‐time surface image of the patient to the baseline reference volume, or ROI, and discrepancies (6 degrees of freedom) are displayed for clinical use. The system operates in two different spatial resolutions: standard and SRS. Standard resolution incorporates a non‐rigid algorithm that is beneficial for non‐rigid treatment sites in regions of the breast or extremities while SRS incorporates a higher resolution with more calculation points along with a more robust algorithm to accommodate for open‐face masks.^16^ The camera has a temporal resolution of 200 frames/sec and its sensitivity can be adjusted with two parameters, Integration time (µs) and Gain (%), to account for difference in reflection produced by different skin tones.^17^

### 2.B Methods

**Fig. 2:**
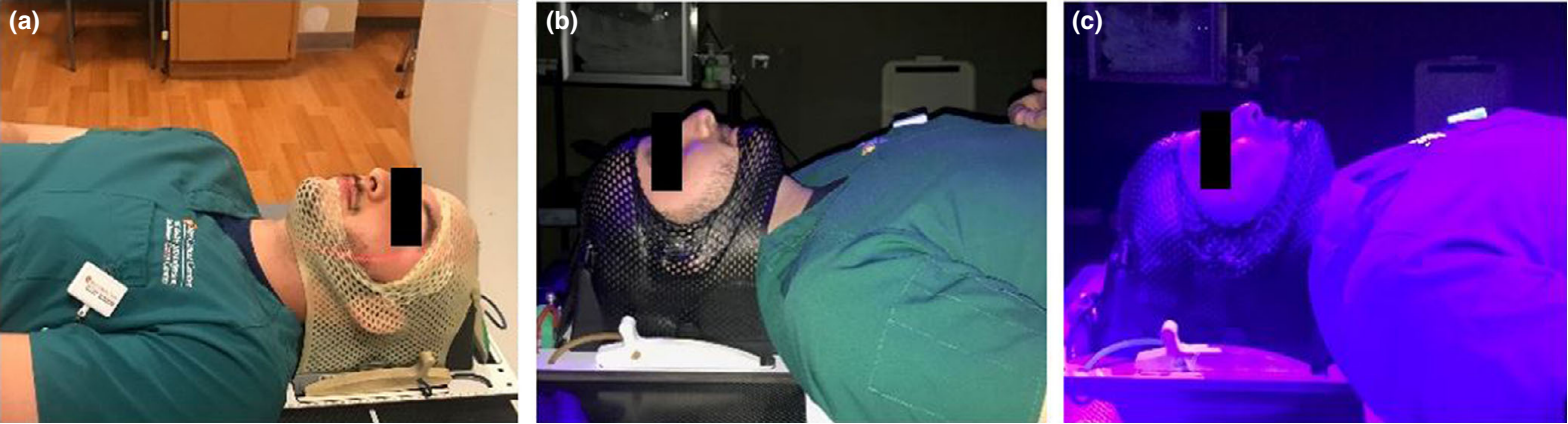
(a) Initial set up of subject in CT room shows the original color of the mask, (b) subject setup in radiation treatment room with mask spray painted matte black, and (c) subject in treatment room with lights from SGRT system (CRAD Catalyst HD).

With Institutional Review Board (IRB) approval, ten healthy human subjects (five female and five male) were used to quantify potential positional deviations due to eye movement and facial expressions with the use of an SGRT system. Individuals were immobilized using precut or modified masks (Orfit’s 5‐point open‐face mask: head, neck and shoulders, Orfit’s 3‐point: head, Orfit’s 3 point open‐face mask, and Brainlab’s SRS immobilization mask) to expose the patient’s face superior to the eyebrows, inferior to the mouth and lateral to the cheeks. All masks were spray painted with a black matte finish to improve skin to mask contrast with the C‐RAD scanner units as shown in Fig. 2. Enhancing the contrast prevents the mask from contributing to positional corrections generated by the SGRT system. We would expect similar efficacy when using a bright colored mask on a darker skin tone however to reduce variables in this study, a common mask surface color was used.

**Fig. 3:**
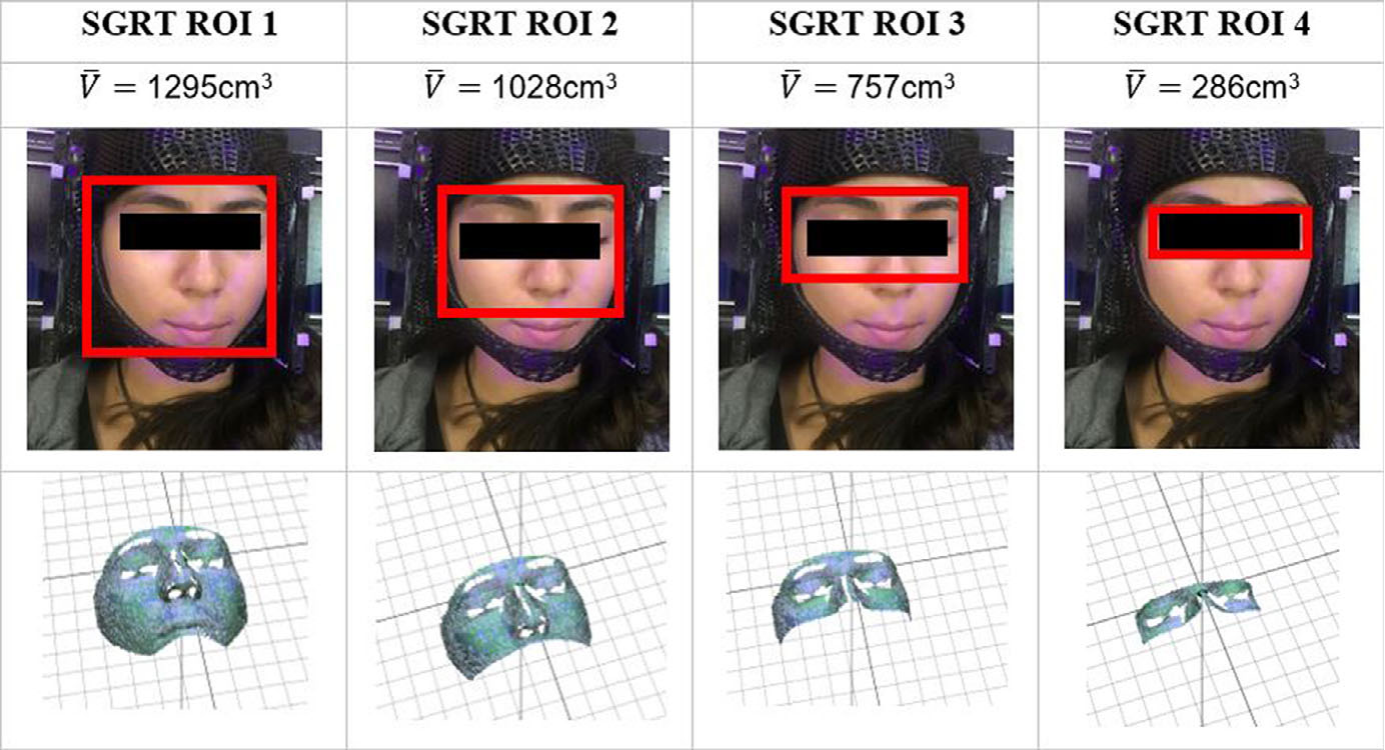
Four discrete SGRT ROIs were chosen based on anatomical features to simulate different mask size openings available in clinics. The average facial surface volume (cm^3^) based on each subject (*n* = 10) is listed. This figure compares the region we aimed to target and how that appeared on the SGRT system display.

To be consistent, individuals were positioned with the Catalyst HD using the SRS resolution (~Time:10,000µs, Gain: 0%), so that the center of their head or “isocenter” was at C‐RAD’s central axis. Camera settings were adjusted to allow for maximum optimization based on skin color. Discrete SGRT ROIs were varied for analysis based on anatomical features and limited in volume based on facial surface area to simulate different size mask openings. The first ROI ($\bar{V}$ = 1295 cm^3^) was lateral to the cheeks, superior to the eyebrows, and inferior to the mouth; the second ROI ($\bar{V}$ = 1028 cm^3^) was lateral to the cheeks, superior to the eyebrows, and superior to the mouth; the third ROI ($\bar{V}$ = 757 cm3) was lateral to the cheeks, superior to the eyebrows, and superior to the tip of nose; and finally the fourth ROI ($\bar{V}$ = 286 cm^3^) included only the eyes and bridge of the nose as shown in Fig. 3. The average surface area of the 4 ROIs (1‐4) were 155 cm^2^, 120 cm^2^, 90 cm^2^, and 34 cm^2^, respectively.

**Fig. 4:**
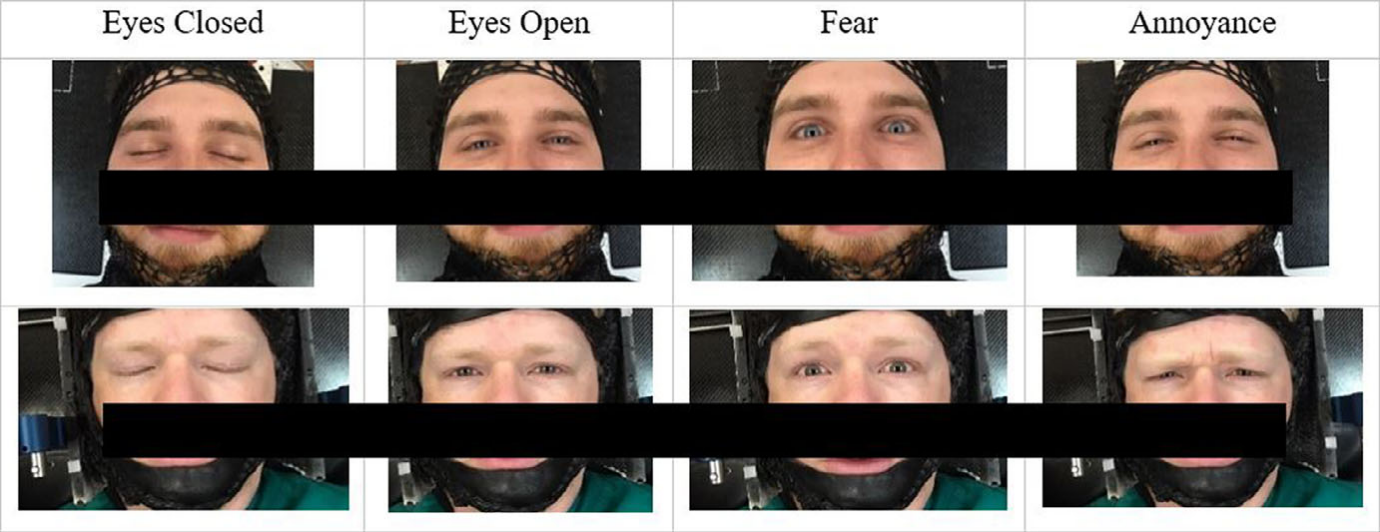
Example of subjects with eyes closed, eyes open, and facial expressions for fear and annoyance.

For each ROI surface, individuals were asked to perform a series of multiple tasks in one sitting, such as open and close their eyes and change facial expressions to simulate an emotional response (see Fig. 4). Since a cancer diagnosis and course of treatment can induce a range of different emotions,^18^ we asked volunteers to simulate fear and annoyance. We considered these to be common emotions for patients as they may be fearful of receiving a radiation treatment or annoyed by the treatment process. Each subject was asked to “close eyes (C1), open eyes, close eyes (C2), express fear, close eyes (C3), express annoyance, and close eyes (C4).” Human subjects were asked to close their eyes between each activity to determine whether patient positioning returned to a steady baseline. Peak translational (Vert, Long, Lat) and angular (Rot, Roll, Pitch) shifts generated by the SGRT system were manually recorded for each task and the total vector shift or total deviation from the isocenter was calculated based on translational shifts using [Eq. (1)].

(1) $$\mathrm{Deviation}=\sqrt{{Vert}^{2}+{Long}^{2}+{Lat}^{2}}$$

To evaluate the efficacy of ROI size and impact of face motion, the average value and standard deviation for discrete peak values of a single task performed were calculated across similar ROI sizes (ROI 1, ROI2, etc.) for all subjects (1, 2, 3, etc.). We calculated a statistical deviation (SD), as seen in Tables 1 and 2, based on the discrete isocenter deviation values recorded for individual tasks using data from all subjects (*N* = 10) in one sitting. Since subjects were asked to close their eyes between each of these tasks, there are four times as many values (*N* = 40). For comparison, this entire process was repeated in standard resolution (~Time:3,000 µs, Gain: 0%).

**Table 1:** Comparison of values for the positional deviations generated by the SGRT system in SRS resolution for eye motion and emotions. Data is presented for ten subjects wearing open‐face masks using four discrete ROIs with C‐RAD’s Catalyst HD motion monitoring system. Closed values for each subject (*N* = 4) are an average of C1, C2, C3 and C4.

SRS Resolution, Isocenter Shift										
Subject	SGRT ROI 1					SGRT ROI 2				
	Volume (cm^3^)	Closed (mm)	Open (mm)	Fear (mm)	Annoyance (mm)	Volume (cm^3^)	Closed (mm)	Open (mm)	Fear (mm)	Annoyance (mm)
1	1512	0.32	0.22	1.94	2.30	1114	0.49	0.36	2.86	1.33
2	1543	0.18	0.10	2.31	0.71	906	0.24	0.14	0.73	0.76
3	1566	0.60	0.36	2.5	1.71	1530	0.30	0.14	1.76	1.51
4	1271	0.30	0.24	1.81	0.42	1016	0.29	0.2	1.9	2.2
5	1034	0.11	0.1	0.54	1.89	970	0.15	0.14	1.53	2.53
6	1542	0.18	0	0.77	0.14	1220	0.12	0.32	0.2	0.42
7	1157	0.22	0.1	0.37	0.55	887	0.14	0.14	0.3	0.51
8	1205	0.51	0.17	0.9	1.68	941	0.14	0.33	1.58	0.99
9	1225	0.43	0.14	2.28	1.86	966	0.35	0.1	2.63	1.12
10	898	0.46	0.17	0.67	1.17	733	0.22	0.41	0.71	0.81
N	10	40	10	10	10	10	40	10	10	10
Average	1295	0.33	0.16	1.41	1.24	1028	0.25	0.23	1.42	1.22
SD	236	0.29	0.10	0.83	0.75	219	0.23	0.12	0.92	0.70

Subject	SGRT ROI 3					SGRT ROI 4				
	Volume (cm^3^)	Closed (mm)	Open (mm)	Fear (mm)	Annoyance (mm)	Volume (cm^3^)	Closed (mm)	Open (mm)	Fear (mm)	Annoyance (mm)
1	862	0.22	0.81	4.5	1.01	253	0.42	0.64	1.4	3.98
2	831	0.55	0.32	1.84	1.5	331	0.18	0.32	0.14	0.55
3	917	0.50	1.04	2.01	1.46	391	0.70	1.08	1.92	2.46
4	762	0.14	0.2	1.3	0.22	326	0.18	0.41	1.82	0.55
5	771	0.27	0.33	0.51	2.73	306	0.23	0.71	0.71	1.57
6	897	0.24	0.14	0.36	0.41	323	0.26	0.42	0.37	0.73
7	640	0.15	0.14	0.83	0.17	243	0.10	1.09	2	2.66
8	688	0.32	0.17	2.97	2.35	254	0.53	1.3	1.88	2.13
9	695	0.29	0.1	2.92	0.45	229	0.28	0.4	1.12	2.11
10	504	0.26	0.68	0.99	1.49	209	0.28	1.57	1.14	1.62
N	10	40	10	10	10	10	40	10	10	10
Average	757	0.30	0.40	1.82	1.18	286	0.32	0.79	1.25	1.84
SD	128	0.27	0.33	1.31	0.89	57	0.29	0.44	0.67	1.08

**Table 2:** Comparison of values for the positional deviations generated by the SGRT system in Standard resolution for eye motion and emotions. Data is presented for ten subjects wearing open‐face masks using four discrete ROIs with C‐RAD’s Catalyst HD motion monitoring system. Closed values for each subject (N = 4) are an average of C1, C2, C3 and C4.

Standard resolution, Isocenter shift										
Subject	SGRT ROI 1					SGRT ROI 2				
	Volume (cm^3^)	Closed (mm)	Open (mm)	Fear (mm)	Annoyance (mm)	Volume (cm^3^)	Closed (mm)	Open (mm)	Fear (mm)	Annoyance (mm)
1	1512	0.36	0.40	4.57	1.40	1114	0.38	0.70	4.84	1.84
2	1543	0.19	0.30	0.14	0.36	906	0.18	0.42	0.42	0.54
3	1566	0.22	0.30	1.92	1.93	1530	0.36	0.33	3.23	2.87
4	1271	0.12	0.14	0.37	0.14	1016	0.29	0.28	1.09	1.03
5	1034	0.18	0.17	0.33	1.22	970	0.15	0.32	0.51	1.57
6	1542	0.18	0.44	0.62	0.35	1220	0.23	0.17	0.24	0.47
7	1157	0.15	0.30	0.24	0.73	887	0.34	0.46	0.67	0.32
8	1205	0.28	0.41	4.35	0.99	941	0.22	1.70	4.67	1.00
9	1225	0.17	0.24	0.42	0.41	966	0.08	0.32	1.60	0.22
10	898	0.35	0.33	1.22	0.75	733	0.21	1.06	0.73	0.77
N	10	40	10	10	10	10	40	10	10	10
Average	1295	0.22	0.30	1.42	0.83	1028	0.25	0.58	1.8	1.06
SD	236	0.14	0.10	1.69	0.56	219	0.13	0.47	1.78	0.82

Subject	SGRT ROI 3					SGRT ROI 4				
	Volume (cm^3^)	Closed (mm)	Open (mm)	Fear (mm)	Annoyance (mm)	Volume (cm^3^)	Closed (mm)	Open (mm)	Fear (mm)	Annoyance (mm)
1	862	0.27	1.09	3.97	0.76	253	1.09	2.54	7.10	2.18
2	831	0.23	0.37	0.41	0.71	331	0.46	1.41	0.97	1.28
3	917	0.35	0.22	1.49	1.55	391	0.50	0.52	1.45	1.73
4	762	0.22	0.24	1.19	0.30	326	0.40	0.65	4.58	0.55
5	771	0.30	0.54	0.73	1.78	306	0.41	2.96	2.94	1.66
6	897	0.47	0.81	0.51	1.75	323	0.29	0.99	2.30	1.03
7	640	0.18	0.36	0.54	0.68	243	0.38	1.02	2.89	8.51
8	688	0.19	2.20	4.20	0.73	254	0.57	3.69	3.68	2.95
9	695	0.12	0.14	1.34	0.17	229	0.15	0.64	3.07	3.02
10	504	0.19	0.64	0.77	0.64	209	0.37	3.51	2.44	2.34
N	10	40	10	10	10	10	40	10	10	10
Average	757	0.25	0.66	1.52	0.91	286	0.46	1.79	3.14	2.53
SD	128	0.16	0.62	1.40	0.58	57	0.42	1.25	1.73	2.25

## 3 Results

### 3.A SRS Resolution

**Fig. 5:**
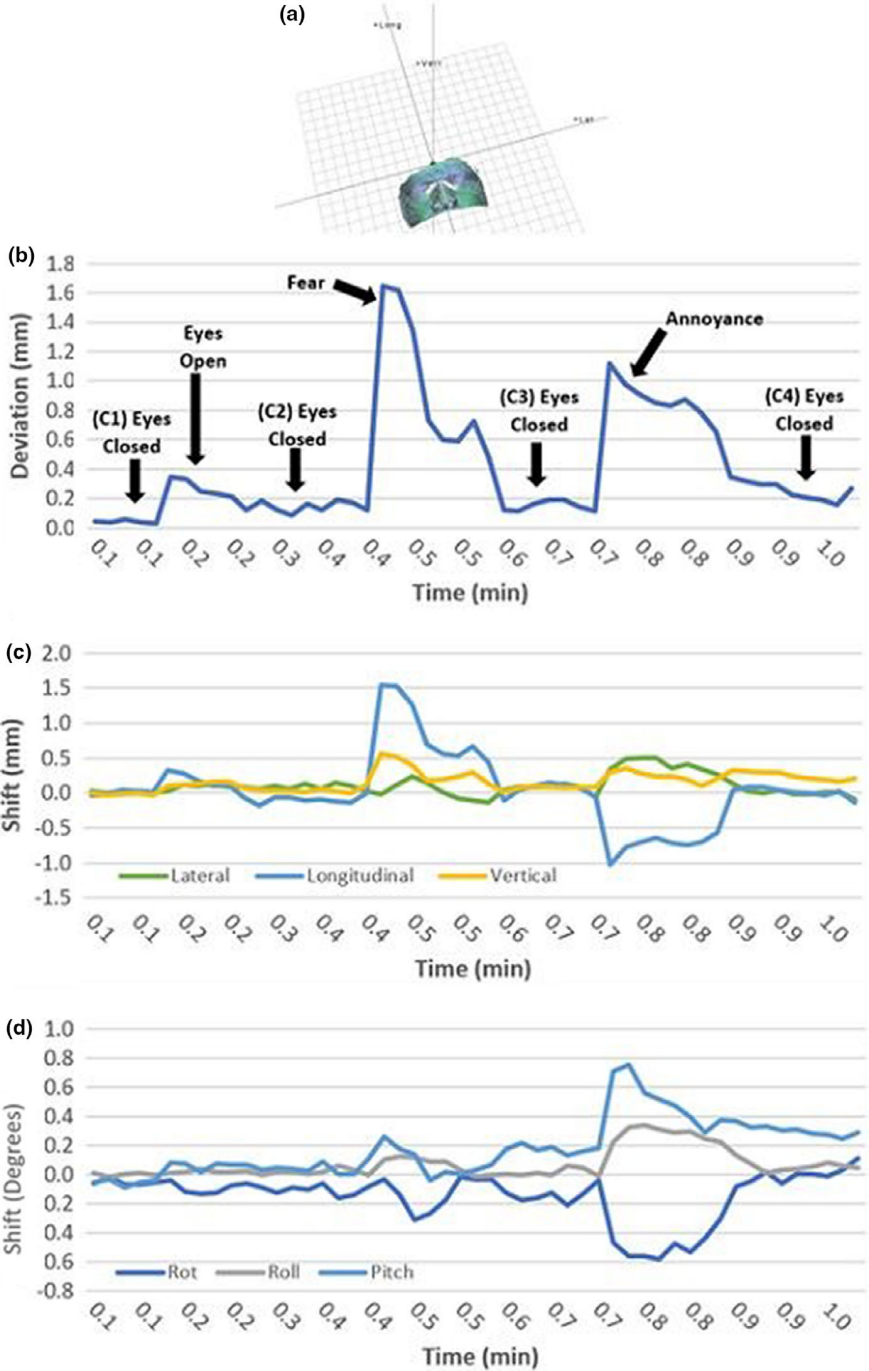
(a) SGRT system (CRAD Catalyst HD) ROI display during data collection; (b) calculated deviation versus time; (c) translational shifts – Lat, Long, and Vert – vs. time; and (d) angular shifts ‐ Rot, Roll and Pitch‐ vs. time for a human subject wearing the open‐face mask. Emotions, fear and annoyance, show the greatest shift in the longitudinal directions (>2 mm).

Figure 5 demonstrates the SGRT data collected for a single ROI in SRS resolution for a human subject in this study. Table 1 shows the SGRT data for each subject and each ROI monitored. On average for all SGRT ROIs, the baseline deviation for eyes closed was 0.3 ± 0.3 mm with a deviation of 0.4 ± 0.4 mm for eyes open. The average deviation due to fear and annoyance was 1.3 ± 0.8 mm for larger ROIs (1 and 2) and 1.5 ± 1.0 mm for smaller ROIs (3 and 4).

**Fig. 6:**
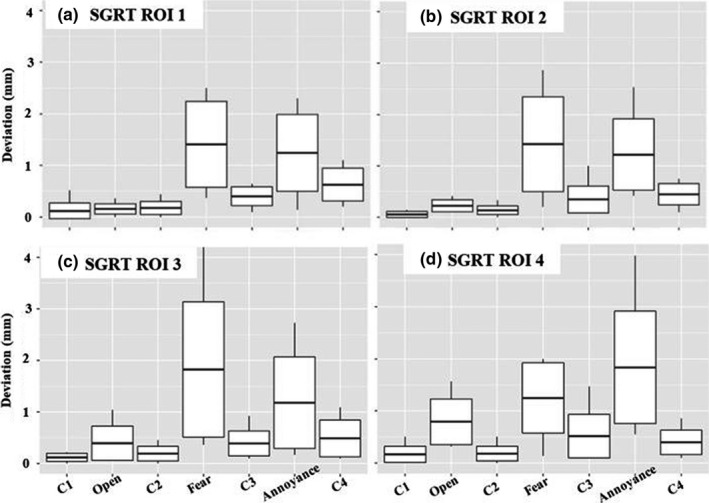
Comparison of positional deviations in SRS resolution for SGRT (a) ROI 1, (b) ROI 2, (c) ROI 3 and (d) ROI 4 for each volunteer (*n* = 10). The center line in each box represents the mean value ($\bar{\mu }$), the ends of the box represents one standard deviation from the mean ($\bar{\mu }\pm \sigma$), and the line extending off the box represents the maximum and minimum range of the data.

Figure 6 shows discrete, maximum positional deviations recorded versus time based on SGRT ROIs for all subjects. The largest deviations are seen when the human subjects are asked to simulate and fear and annoyance and this is amplified in the smaller ROIs (3 and 4) when compared to the larger ROIs (1 and 2).

**Fig. 7:**
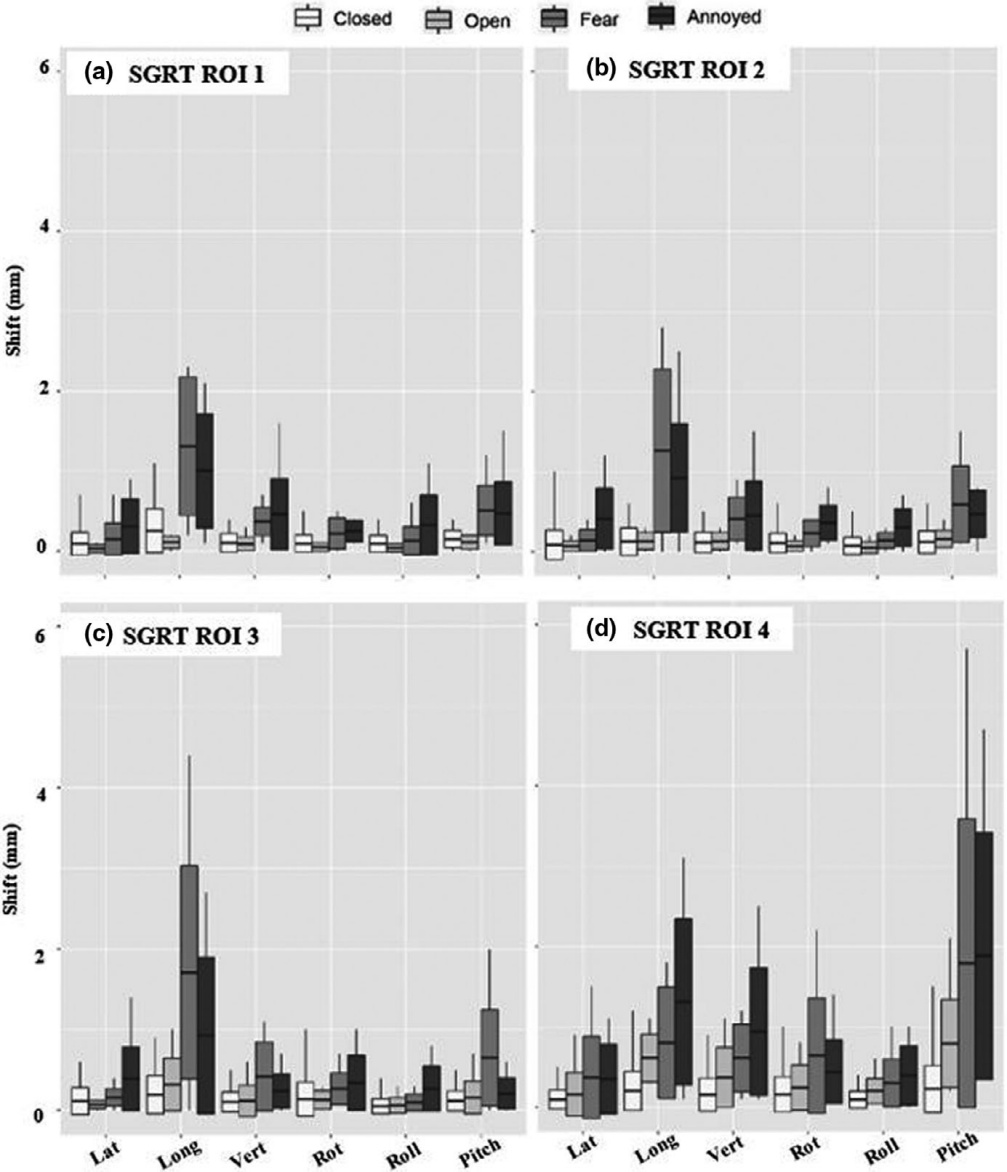
The absolute translational and angular shifts in SRS resolution for each task: Closed (*n* = 40), Open (*n* = 10), Fear (*n* = 10) and Annoyance (*n* = 10).

Figure 7 shows the individual translational and angular shifts observed based on SGRT ROIs for all subjects. Large longitudinal shifts are observed for fear and annoyance for all SGRT ROIs and a significant shift in the pitch direction is amplified for ROI 4.

**Fig. 8:**
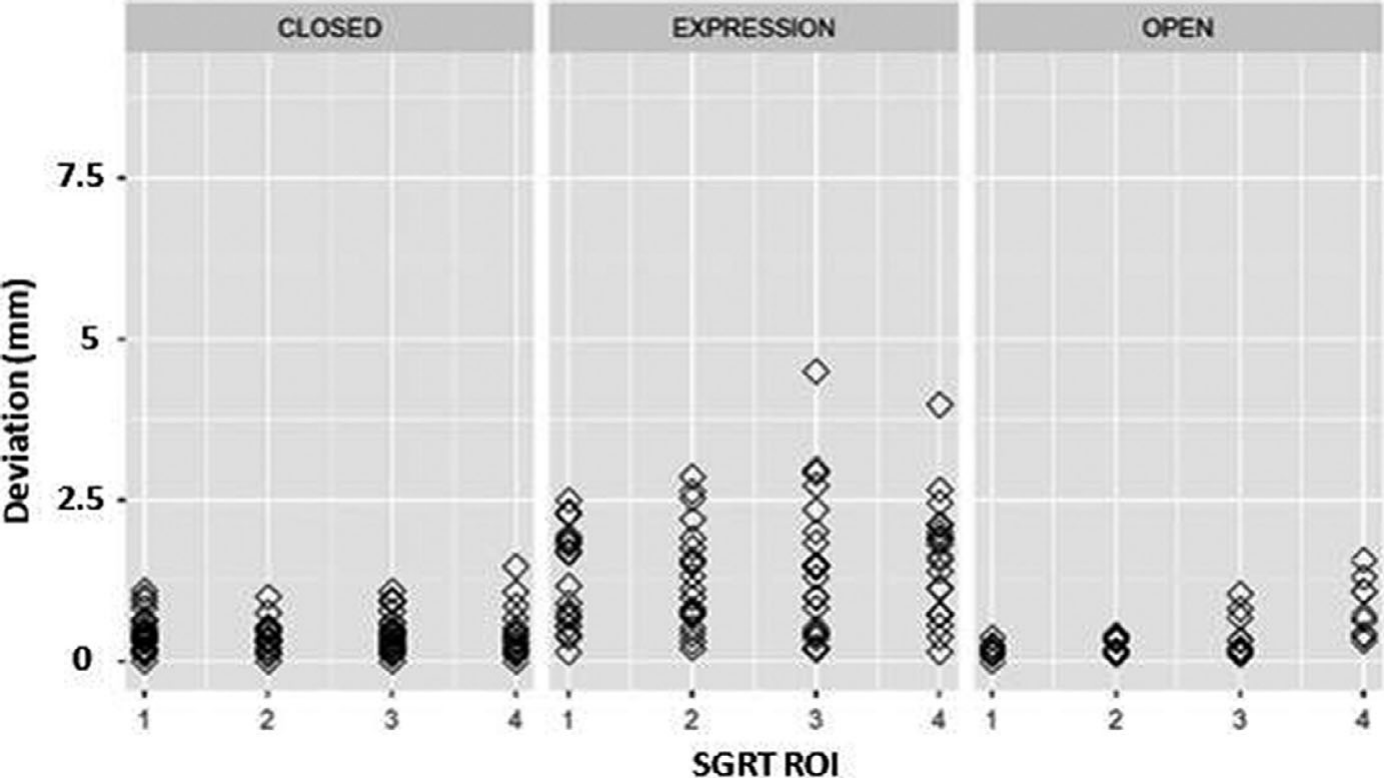
SGRT recorded deviations for eyes closed, eyes open and expression (fear and annoyance) organized by ROI size.

**Fig. 9:**
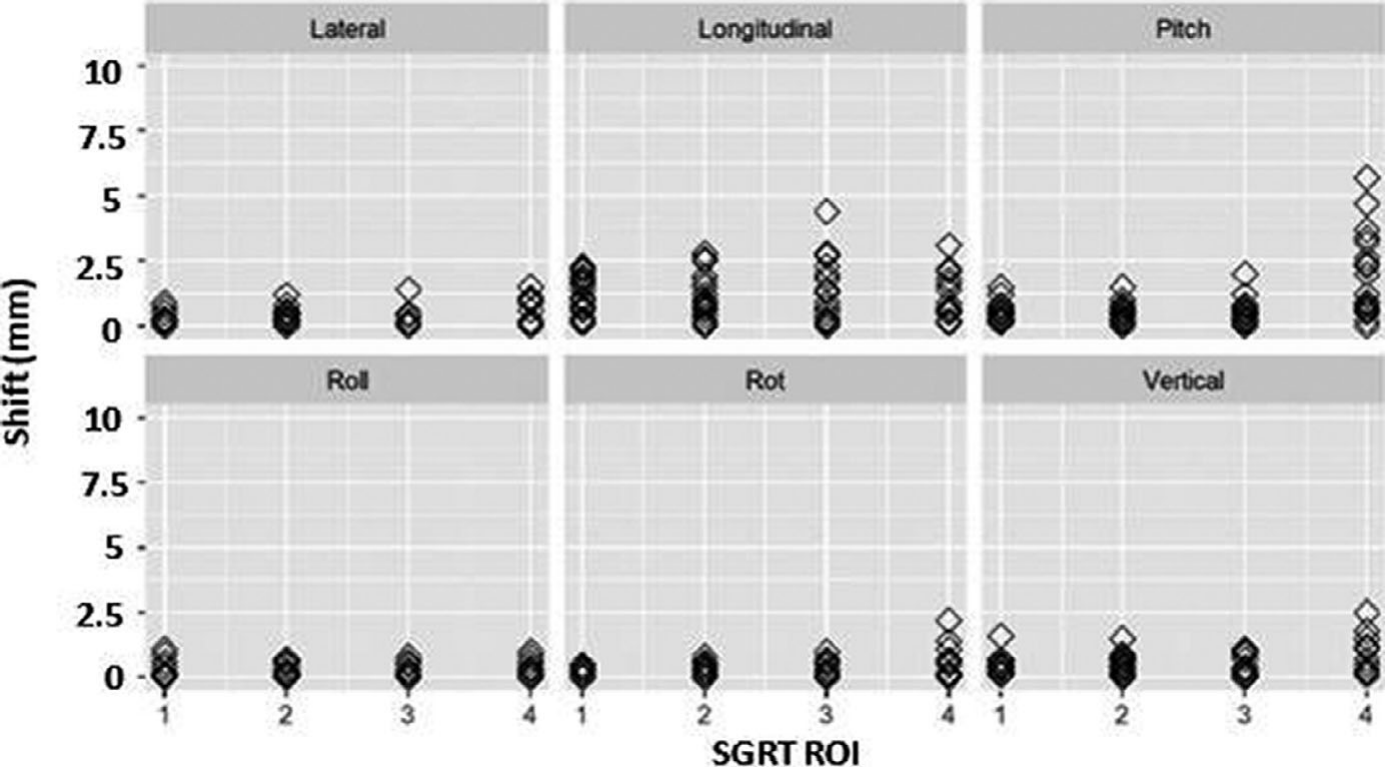
Translational and angular shifts for expression (fear and annoyance).

Figures 8 and 9 demonstrate deviation and translational and angular deviations however they differ from the previous figures as they combine fear and annoyance under one title ‘expression.’

### 3.B Standard resolution

Table 2 shows the SGRT data for each human subject and each ROI monitored. On average for all SGRT ROIs, the baseline deviation for eyes closed was 0.3 ± 0.3 mm and 0.8 ± 0.9 mm for eyes open. The average shift due to fear and annoyance was 1.3 ± 1.3 mm for larger ROIs (1 and 2) and 2.0 ± 1.8 mm for smaller ROIs (3 and 4). 

**Fig. 10:**
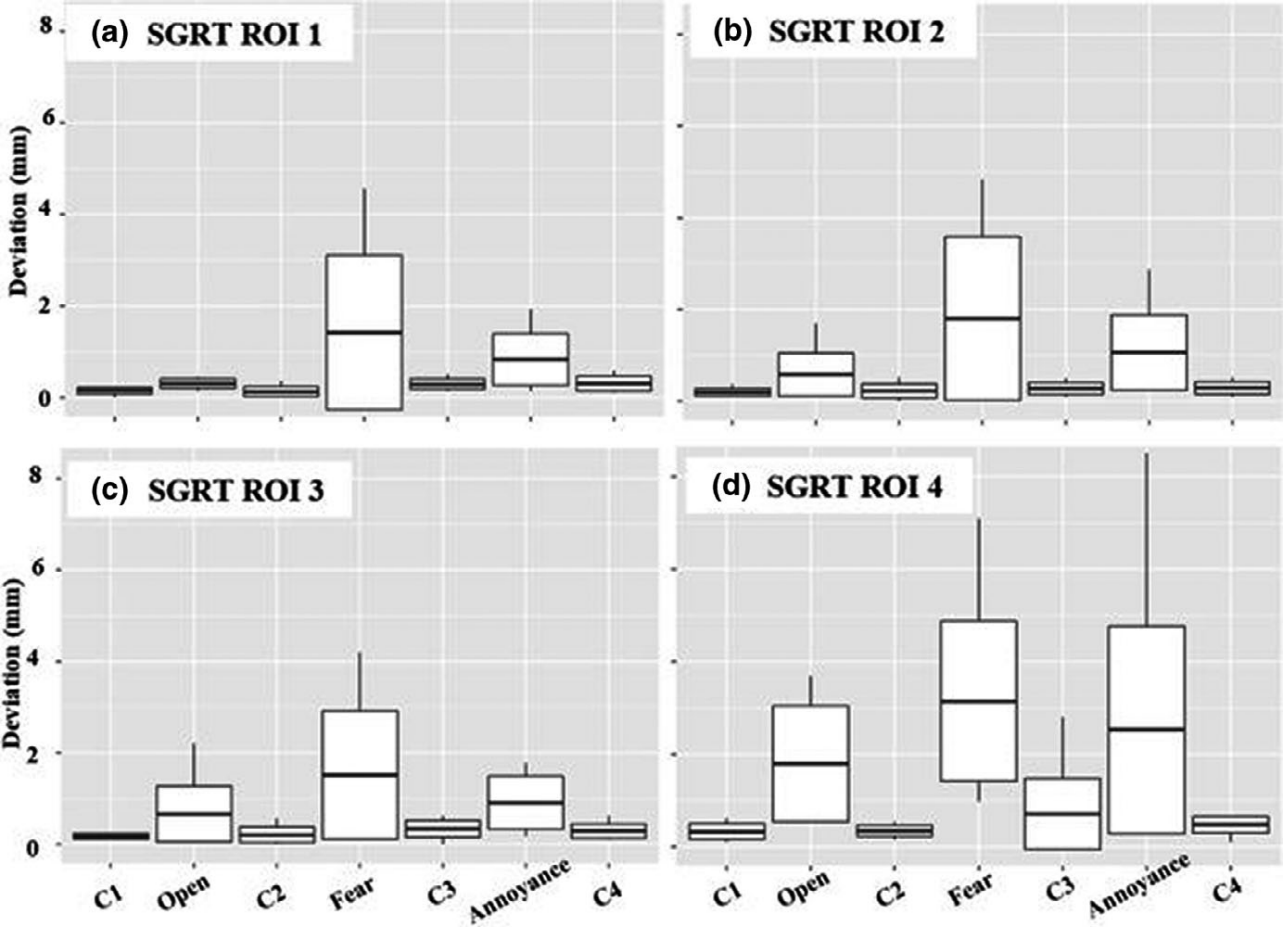
Comparison of positional deviations in Standard resolution for SGRT (a) ROI 1, (b) ROI 2, (c) ROI 3 and (d) ROI 4 for each volunteer (*n* = 10). The center line in each box represents the mean value (μ ®), the ends of the box represents one standard deviation from the mean (μ ® ± σ), and the line extending off the box represents the maximum and minimum range of the data.

Figure 10 shows discrete, maximum positional deviations recorded over time based on SGRT ROIs for all subjects. Similar to the results for SRS resolution, the largest deviations are still seen when the human subjects are asked to simulate fear and annoyance.

**Fig. 11:**
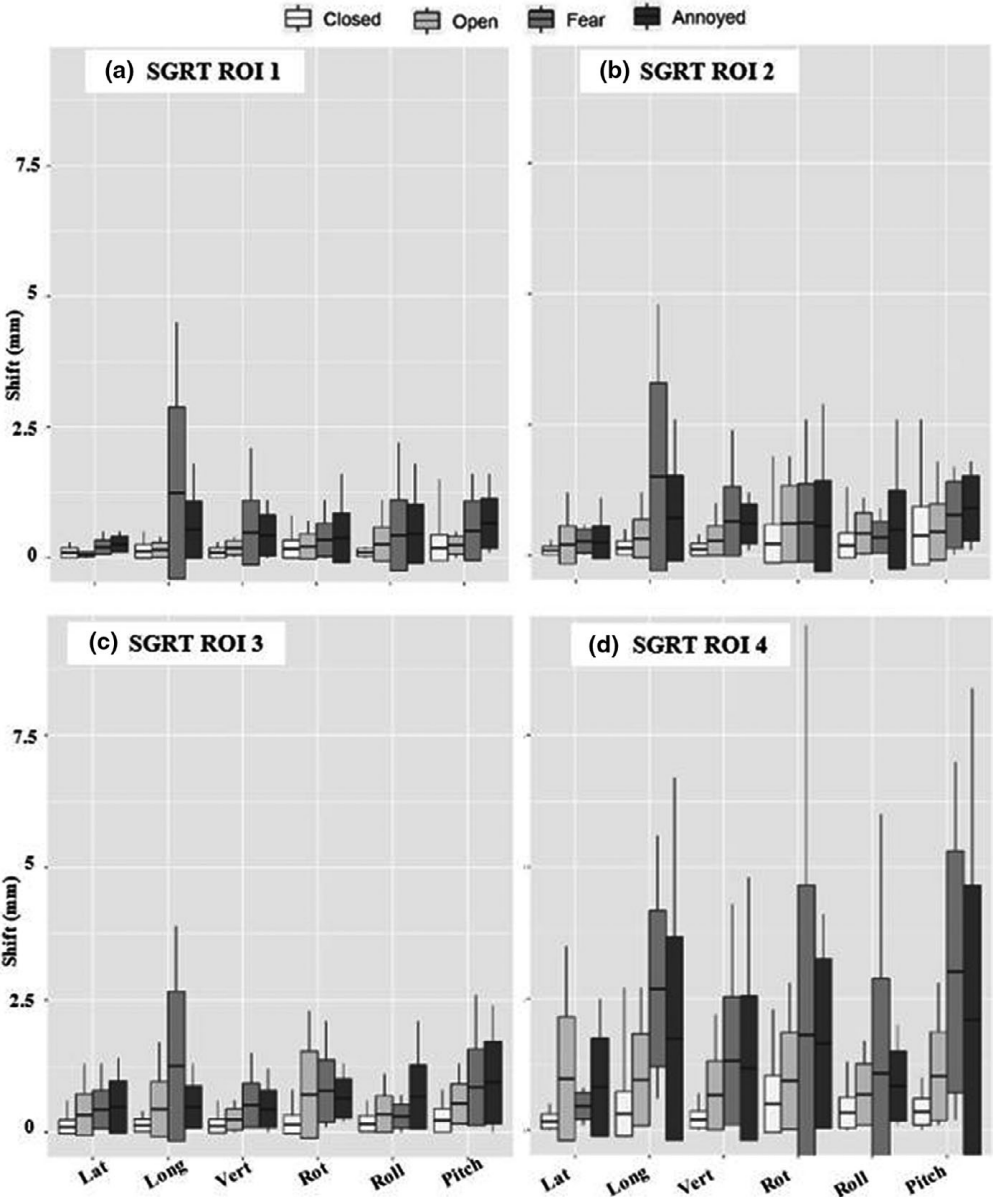
The absolute translational and angular shifts in standard resolution for each task: Closed (*n* = 40), Open (*n* = 10), Fear (*n* = 10) and Annoyance (*n* = 10).

Figure 11 shows the individual translational and angular shifts observed based on SGRT ROIs for all subjects. Similar to results in SRS resolution, large longitudinal shifts are observed for fear and annoyance for all SGRT ROIs however the smallest ROI (4) shows amplification in multiple directions.

**Fig. 12:**
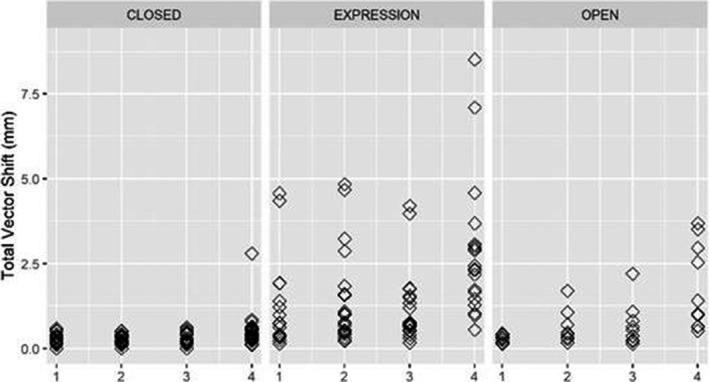
SGRT recorded deviations for eyes closed, eyes open and expression (fear and annoyance) organized by ROI size.

**Fig. 13:**
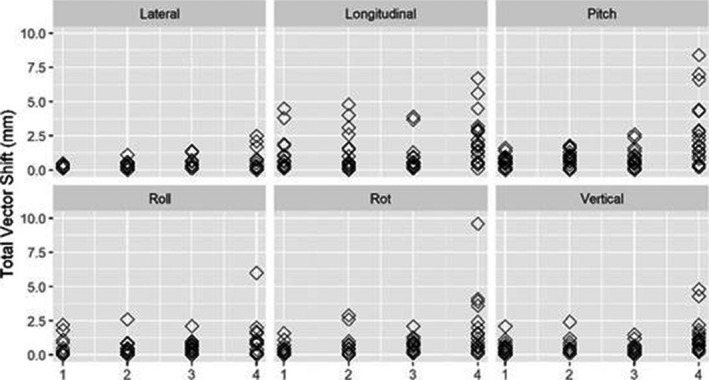
Translational and angular shifts for expression (fear and annoyance).

Figures 12 and 13 demonstrate deviation and translational and angular deviations however they differ from the previous figures as they combine fear and annoyance under one title ‘expression.’

## 4 Discussion

This study demonstrated that false positional corrections can be registered by an SGRT system and amplified based on spatial resolution and ROI size, for patients who change facial expressions. It is important to identify and quantify potential sources of error generated by the SGRT system for clinics who want to employ this system. Understanding its limitations will help us better understand how to interpret the data it provides to monitor intrafraction motion or use it as a safety interlock to halt a treatment as patients exceed a specified threshold.

It is entirely possible that patients will not express exaggerated forms of facial expression during treatment such as was simulated here. Li et al.^4^ reported that patients may be unlikely to have significant changes in facial expressions, such as smiling or squeezing of the nose, but if they do, we will see deviations return to a baseline after the patient has relaxed. In this study, we assumed that subjects remained in original treatment positions when performing tasks, as the isocenter shift generally returned to baseline after they relaxed and closed their eyes, within 1mm for standard and SRS specific resolutions. Li et al.^4^ also mentioned that minor facial expressions, such as blinking, would not cause a noticeable change (<1mm) in isocenter deviation. We found this to be true for our largest ROI in standard resolution and the two largest ROIs in SRS resolution. It was evident that as the SGRT ROI size decreased, deviations and shifts for minor and major expressions became amplified (Figs. 6 and 8 for SRS and 10 & 12 for Standard). The larger SGRT ROIs may provide smaller positional corrections as they eliminate noise with the inclusion additional topographic facial information such as the eyebrows and nose. It is important to note that monitoring the entire face with an open mask has been recommended to minimize registration uncertainties.^14^ Finally, we do not believe there would be any practical difference between our simulated tracking methods versus actual masks with various openings since the surface imaging system only monitors discrepancies within the ROI. Our recommendation is to only track the patient’s surface since the mask can move independently.

The two operational modes (SRS and standard resolution) showed no statistically significant differences in deviations shifts for eyes closed or emotional response (*P* = 0.5744 and *P* = 0.901, Wilcoxon rank‐sum) but a difference was seen for eyes open (*P* = 0.1643, Wilcoxon rank‐sum). The spread of values for shifts due to facial expressions appeared larger in standard resolution than in SRS, specifically in the longitudinal and pitch directions (Figs. 9 and 13). This may be due to an individual raising their eyebrows, creasing their forehead or movement of the nose or mouth. These errors may potentially be reduced with the incorporation of a bite block or additional immobilization points of contact.

Relying on any technology or immobilization device can only go so far as patients are left alone in the treatment room during delivery. It is important to emphasize the importance of patient education when using SGRT in our clinics. It is common for therapists to coach and provide instructions to patients on what to expect during the treatment process. This has been demonstrated to reduce anxiety and improve preparedness for treatment.^19^ Therapists should ask patients to remain relaxed with their eyes closed throughout the duration of treatment simulation and delivery.

Subject 7 (Table 2, SGRT ROI 4) registered with a shift greater than 8mm in their annoyance response, demonstrating the possibility of a major treatment deviation using SGRT. Up to 1.1mm isocenter shift was observed for a relaxed facial expression with eyes closed in SRS resolution for the two largest and most practical ROIs (average facial surface area ≥ 120 cm2) for SGRT. This 1mm shift should be considered relative to the planning target volume (PTV) and in the overall SGRT tolerance delivery. The PTV for SRS may be millimeters in size while head and neck tumors are often larger. If positional discrepancies > 1 mm are observed by the SGRT system or if the facial area monitored becomes compromised, we suggest repeating steps for radiographic positional verification. Single target SRS plans may or may not include PTV setup margins typically, depending on the overall delivery accuracy of the specific system used to deliver the radiation. Multi target, single isocenter SRS plans have been observed to be susceptible to compromised target coverage as a result of uncorrected patient motion and typically have some margin applied.^20^

While this study did not directly observe changes of facial expression with couch rotation, the mechanical accuracy of the system needs to be factored in for patient treatment angles as well. It is important to remember that increasing couch angles can decrease the facial surface observed by the SGRT system and compromise its accuracy.^14^ Clinics should commission SGRT systems at all couch angles with a phantom to verify accuracy and individual patient QA tests should check if couch rotations compromise the facial area monitored during treatment.

## 5 Conclusion

This study demonstrated that an SGRT system can generate false positional corrections when patients change facial expressions. These errors are amplified with lower spatial resolutions and smaller SGRT ROIs. It is important to understand the limitations of the SGRT ROI monitored and to understand when additional radiographic imaging may be warranted. These errors may stem from a patient creasing their forehead or by motion of the eyebrows or nose. This paper shows the importance of greater topographic anatomical information (eyebrow and nose regions) to reduce these errors. This should be considered in the overall tolerance in planning and delivery in conjunction with surface imaging for setup and active patient surveillance.
